# Granulocyte Colony-Stimulating-Factor-Producing Bladder Carcinoma: A Case Report and Literature Review

**DOI:** 10.7759/cureus.83447

**Published:** 2025-05-04

**Authors:** Kaori Yamashita, Keita Yoshida, Satoshi Kubota, Tetsushi Sakamoto, Takahiro Shiseki, Hirotaka Uematsu, Tadao Nakazawa, Masashi Inui

**Affiliations:** 1 Department of Urology, Tokyo Women’s Medical University Yachiyo Medical Center, Yachiyo, JPN; 2 Department of Pathology, Tokyo Women’s Medical University Yachiyo Medical Center, Yachiyo, JPN; 3 Department of Urology, Graduate School of Medicine, Chiba University, Chiba, JPN

**Keywords:** bladder, carcinoma, granulocyte colony-stimulating factor, leukemoid reaction, white blood cell

## Abstract

Granulocyte colony-stimulating factor (G-CSF)-producing bladder carcinoma has an aggressive clinical course. We report a case of G-CSF-producing bladder carcinoma in a 78-year-old Japanese man who had a bladder tumor with a diameter of 38 mm. Transurethral resection of the bladder tumor was performed. Pathological examination revealed a high-grade muscle-invasive urothelial carcinoma (pT2). The patient had three courses of neoadjuvant chemotherapy with a combination of gemcitabine and carboplatin and thereafter underwent robot-assisted radical cystectomy. The surgically resected bladder specimen contained a highly invasive tumor with necrosis. The tumor cells showed marked cytological atypia with brisk mitosis. The tumor had metastasized to a regional lymph node. Therefore, we pathologically diagnosed high-grade invasive urothelial carcinoma, stage pT3b pN1. Thirty-six days after radical cystectomy, computed tomography revealed local recurrence and para-aortic and bilateral common iliac lymph node metastasis (white blood cell count had increased to 46,970/µL). Fifty-seven days after radical cystectomy, the white blood cell count further increased to 83,700/µL, and the serum G-CSF level was 186 pg/mL (normal range, 10.5-57.5 pg/mL). G-CSF immunohistochemistry was performed, and diffuse cytoplasmic positivity for G-CSF was verified. Therefore, we considered that a leukemoid reaction had occurred because of G-CSF-producing bladder carcinoma. Seventy-seven days after radical cystectomy, the patient died because of the recurrence of bladder carcinoma (white blood cell count: 85,660/µL).

If a clinician observes bladder carcinoma with an abnormal number of white blood cells despite the lack of a hematopoietic neoplasm or inflammation, G-CSF-producing bladder carcinoma should be considered.

## Introduction

Granulocyte colony-stimulating factor (G-CSF) is a cytokine involved in the maturation and mobilization of bone marrow neutrophils [1]. Clinicians often use G-CSF to treat patients with chemotherapy-induced neutropenia. However, G-CSF-producing tumors are relatively rare and induce an abnormally high white blood cell (WBC) count. Some cases of G-CSF-producing tumors cause a leukemoid reaction (i.e., leukocyte count >50,000/µL). Block et al. reported the first case of G-CSF-producing bladder carcinoma in 1973 [2]. Since then, to the best of our knowledge, approximately 41 reports of G-CSF-producing bladder carcinoma have been documented. Kohno et al. reported that the most frequent site of G-CSF-producing carcinoma is the lung (94 of 420 cases; 22.4%), followed by the urinary bladder and upper urinary tract (57 of 420 cases; 13.6%), stomach and duodenum (38 of 420 cases; 9%), esophagus (30 of 420 cases; 7.1%), liver (23 of 420 cases; 5.5%), pancreas (20 of 420 cases; 4.8%), uterus (20 of 420 cases; 4.8%), and other areas (138 of 420 cases; 32.8%) [3]. Therefore, the bladder is a common site of G-CSF-producing carcinoma.

No established treatment exists for G-CSF-producing bladder carcinoma. In addition, Muramatsu-Maekawa et al. reviewed 40 cases of G-CSF-producing bladder carcinoma and noted that 54.3% (19 of 40 patients) were dead by a median survival time of 6 months [4]. Therefore, G-CSF-producing bladder carcinoma has an early progression and extremely poor prognosis [1,4].

We had a patient with a case of leukemoid reaction due to G-CSF-producing invasive bladder carcinoma. The patient died 77 days after radical cystectomy. Patients with G-CSF-producing bladder carcinoma are more likely to have a pathologically invasive lesion at the time of the initial diagnosis and an aggressive clinical course. In this paper, we describe our experience treating a patient with G-CSF-producing bladder carcinoma. In addition, we review the recent available literature on patients with G-CSF-producing bladder carcinoma. We also provide a flowchart for patients with leukemoid reactions and suggest a treatment for G-CSF-producing bladder carcinoma.

## Case presentation

**Figure 1:**
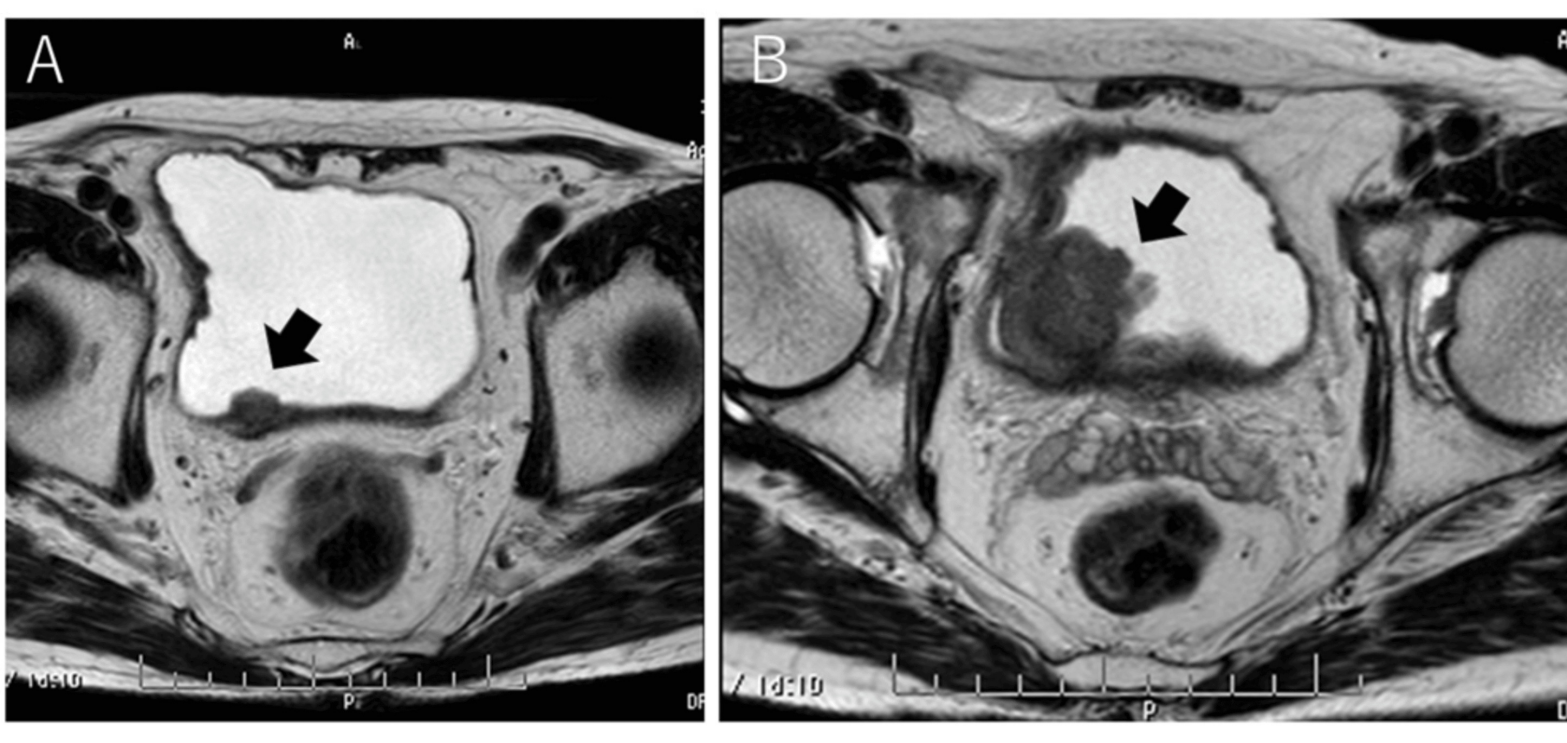
Magnetic resonance imaging findings (A) The bladder tumor (arrow) with a diameter of 15 mm at the initial visit. (B) Three months after his initial visit, the bladder tumor (arrow) has a diameter of 38 mm.

In December 2023, a 78-year-old Japanese man visited our hospital with macrohematuria. Magnetic resonance imaging (MRI) revealed a bladder tumor with a diameter of 15 mm (Figure 1A). We recommended he undergo transurethral resection of bladder tumor (TUR-Bt). However, he rejected the operation at that time.

At three months after his first diagnosis of the bladder tumor, MRI revealed the bladder tumor had a diameter of 38 mm (Figure 1B). The laboratory findings revealed anemia and leukocytosis. The blood analysis findings were as follows: hemoglobin level was 13.8 g/dL; WBC count was 18,680/µL with predominant polymorphonuclear cells on differential count (87.1% neutrophils, 7.3% lymphocytes, 3.3% monocytes, 1.4% eosinophils, 0.9% basophils, 1.0% myelocyte); and C-reactive protein level was 0.46 mg/dL (Table 1). Computed tomography (CT) revealed no metastases to lymph nodes or other organs. His clinical stage was cT3N0M0. In April 2024, TUR-Bt was performed. The patient was pathologically diagnosed with high-grade muscle-invasive urothelial carcinoma (at least stage pT2). Given this patient’s T stage of pT2, we decided that a second TUR was not a treatment option.

**Table 1:** Laboratory investigations of the patient

Parameter	Finding	Normal range
White blood cell	18680/mL	4000–8600/mL
Neutrophils	87.1 %	45–74 %
Lymphocytes	7.3 %	27–47 %
Eosinophils	1.4 %	0–6 %
Basophils	0.9 %	0–2 %
Monocytes	3.3 %	2–8 %
Myelocyte	1.0 %	0 %
Hemoglobin	13.8 g/dL	14–18 g/dL
Platelet	20.8 ×10^4 ^/mL	15–18 ×10^4^/mL
Total protein	6.8 g/dL	6.5–8.2 g/dL
Albumin	4.4 g/dL	3.8–5.1 g/dL
Total bilirubin	0.8 mg/dL	0.1–1 mg/dL
Aspartate transaminase	16 U/L	13–33 U/L
Alanine transaminase	10 U/L	6–30 U/L
Alkaline phosphatase	77 U/L	38–113 U/L
Blood urea nitrogen	17.6 mg/dL	8–20 mg/dL
Creatinine	0.93 mg/dL	0.69–1.06 mg/dL
Sodium	143 mEq/L	135–145 mEq/L
Potassium	3.6 mEq/L	3.4–4.9mEq/L
Chloride	109 mEq/L	98–108mEq/L
C-reactive protein	0.46 mg/dL	0–0.33mg/dL

The patient's estimated glomerular filtration rate before undergoing neoadjuvant chemotherapy was 34.3 mL/min/1.73 m2, indicating he had chronic kidney disease. We selected carboplatin and gemcitabine, despite cisplatin and gemcitabine as the neoadjuvant chemotherapy. After three courses of neoadjuvant chemotherapy, CT imaging revealed that the bladder tumor had shrunk to a diameter of 25 mm. However, the bladder tumor remained; therefore, he subsequently underwent robot-assisted radical cystectomy (RARC) with the creation of an ileal conduit in August 2024.

**Figure 2:**
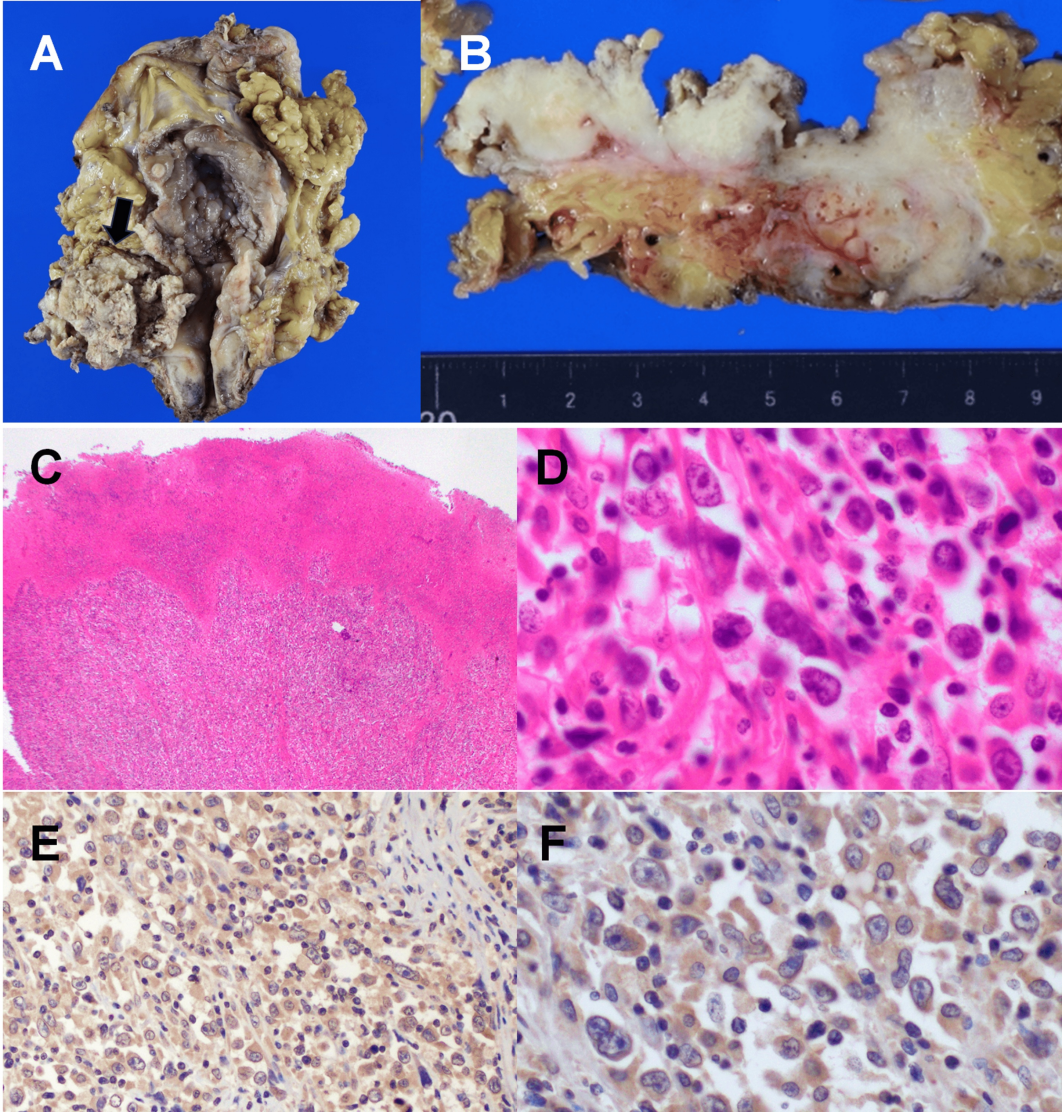
Macroscopic and microscopic findings Resected specimen of the bladder tumor. (A) The gross examination shows an approximately 5-cm bladder tumor in the right lateral wall that occupies the right ureteral orifice. (arrow). (B) On the cut surface, the tumor has entirely penetrated the wall. (C) Microscopic findings reveal neoplastic cells arranged in a solid pattern with necrosis (H&E; magnification, ×50). (D) High-power magnification reveals enlarged nuclei with prominent nucleoli and brisk mitotic figures (H&E; magnification, ×200 and ×400). (E and F) Immunohistochemistry shows that the cytoplasm of the tumor cell is diffusely positive for G-CSF. H&E, hematoxylin and eosin; G-CSF, granulocyte colony-stimulating factor

In the surgical specimen, a bulky mass, approximately 5 cm, had grossly replaced all layers of the right lateral wall of the urinary bladder (Figures 2A, 2B). The tissues were routinely processed, cut, deparaffinized, and stained with hematoxylin and eosin. Immunohistochemistry (IHC) was carried out using the Leica BOND-MAX immunostainer (Leica Biosystems, Newcastle, UK). Microscopic observation revealed that solid tumor cell clusters infiltrated the entire wall and the surrounding adipose tissue with necrosis (Figure 2C). These tumor cells had an oval to polygonal shape with marked cytological atypia, with brisk mitosis intermingled with variable degrees of inflammatory cells (Figure 2D). IHC revealed diffuse positive immunostaining for pancytokeratin markers (e.g., AE1/AE3). The tumor had metastasized to a lymph node around the right obturator nerve. Overall, the pathological diagnosis was invasive urothelial carcinoma, high-grade, INFa, ypT3a, LVI0, u-rt0, u-lt0, ur0, yp withN1 (1/9) (Figure 2).

After the RARC, he had a paralytic ileus and had not eaten for one month. At 36 days after RARC, the WBC count reached 46,970/µL, but the patient had no fever or an increase in the C-reactive protein level. Therefore, infection was not suspected. We consulted a hematologist, but myeloproliferative disorders, such as leukemia, were not indicated. CT revealed concomitant local recurrence and para-aortic and bilateral common iliac lymph node metastasis. Second-line chemotherapy, such as pembrolizumab, was considered for this patient. However, the disease was progressive, and his condition worsened. The patient could not take the second-line chemotherapy.

CT revealed bilateral hydronephrosis due to local recurrence and para-aortic lymph node metastasis. Fifty-seven days after RARC, the WBC count reached 83,700/µL, and the patient`s serum G-CSF level was 186 pg/mL (normal range, 10.5-57.5 pg/mL). We speculated that the serum G-CSF level was elevated by G-CSF-producing bladder cancer. We outsourced G-CSF immunohistochemistry analysis of the surgical specimens. Immunostaining was performed using a mouse monoclonal antibody against G-CSF (clone 4-12-2; dilution 1:50; Immuno-Biological Laboratories Co., Ltd., Fujioka, Japan). As a consequence, the cytoplasm of the tumor cells was diffusely positive for G-CSF (Figures 2E, 2F). Thus, the patient’s leukemoid reaction was caused by a G-CSF-producing bladder carcinoma. Seventy-seven days after RARC, he died because of the recurrence of bladder carcinoma (WBC count was 85,660/µL).

## Discussion

Based on our investigation, seven case reports have been published since 2013 [4-10], a time when programmed death-1 receptor/ligand (PD-1/PD-L1) antibodies such as pembrolizumab began to transform the landscape of oncology [11]. We have summarized the literature review of the eight recent case reports on G-CSF-producing bladder carcinoma in Table 2, including our report. With regard to pathological specimens, all eight patients’ specimens showed invasive urothelial carcinoma, five of the eight patients had high-grade urothelial carcinoma, and three of the eight patients had poorly differentiated urothelial carcinoma. Therefore, G-CSF-producing bladder carcinoma pathologically may be an aggressive urothelial carcinoma.

**Table 2:** Summary of recent reported clinical cases of bladder carcinoma with leukemoid reaction WBC, white blood cell; G-CSF, granulocyte colony-stimulating factor; UC, urothelial carcinoma; RC, radical cystectomy; PEM, pembrolizumab

Author (reference no., publication year)	Age (y)/sex	Pathology	Peak WBC (/µL)/neutrophils (%)	G-CSF (pg/mL)	Metastasis at the initial visit/stage	Treatment	Outcome
Nagasaki et al. [5] (2023)	62/M	Poorly differentiated invasive UC	90,600 (at the initial visit)	498	Prostate, heart, and subcutaneous mass on the chest wall	None	Died 49 days after his initial visit because of heart failure
Muramatsu-Maekawa et al.[4] (2020)	53/M	High-grade invasive UC	18,800 (at the initial visit)	None	None/pT4aN0M0	Four courses of chemotherapy (gemcitabine and carboplatin), RC, PEM	One-year survival after the initial treatment with PEM
Morinaga et al. [6](2019)	67/F	Highly invasive UC with squamous cell differentiation	17,100 (at the initial visit)/ 79.8	None	Iliac lymph node, colon	None	Died six months after her initial visit
Kato et al. [7](2016)	38/F	Invasive, poorly differentiated UC	10,7000 (at the initial visit)/91.5	77.1	Multiple lymph nodes, multiple bone	One course of chemotherapy (gemcitabine and cisplatin)	Died 15 days after the initiation of chemotherapy
Kumar et al. [8](2014)	39/F	Poorly differentiated high-grade transitional cell carcinoma	57,800 (at the initial visit) /74.8	406.6	None/pT4aN0M0	RC	Died 5 months after RC
Shapiro et al. [9](2014)	43/M	High-grade transitional cell carcinoma	95,200 (before RC)/77	None	None/pT3bN0M0	RC	Died 57 days after RC
He et al. [10](2013)	64/M	Invasive uroepithelium carcinoma of the bladder	58,400 (before RC)/94	4032	None	RC	Died 3 months after leaving the hospital
Our case (2025)	78/M	High-grade invasive urothelial carcinoma	87,300 (before death)/94.5	186	None/pT3apN1M0	RC	Died 77 days after RC

**Figure 3:**
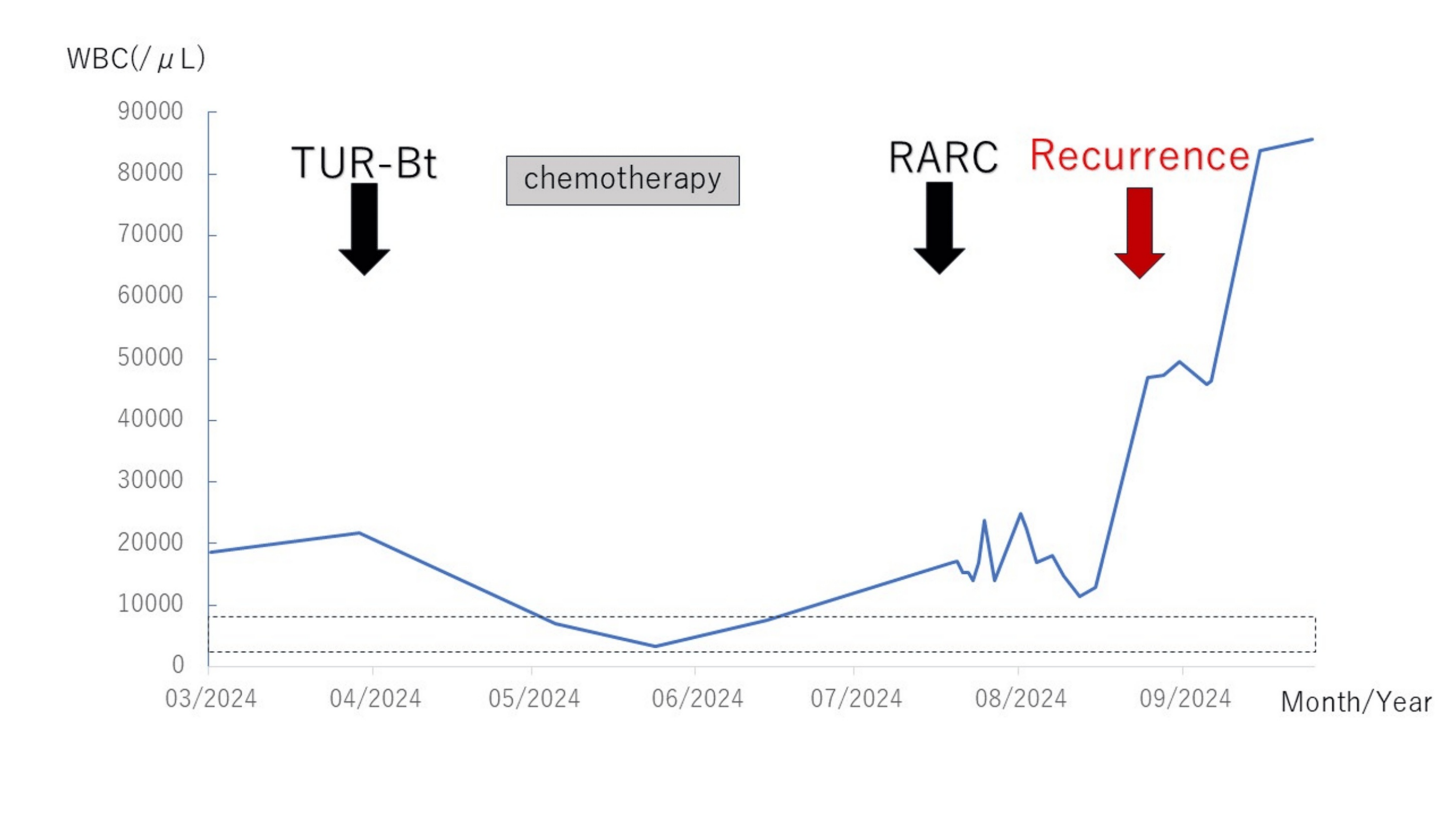
The WBC count (/µL) during the clinical course A normal WBC count ranges from 4000 to 8600/µL (short-dashed lines). WBC, white blood cell; TUR-Bt, transurethral resection of bladder tumor; RARC, robot-assisted radical cystectomy

The number of WBCs may depend on the size of a G-CSF-producing bladder carcinoma because the number of WBCs decreased after the patient underwent RARC and increased again when the cancer recurred. The maximum number of WBCs occurred before the patients died (Figure 3); the other authors reported the same tendency in the WBC count [4-10]. Therefore, the number of WBCs may serve as a tumor marker for G-CSF-producing bladder carcinoma.

**Figure 4:**
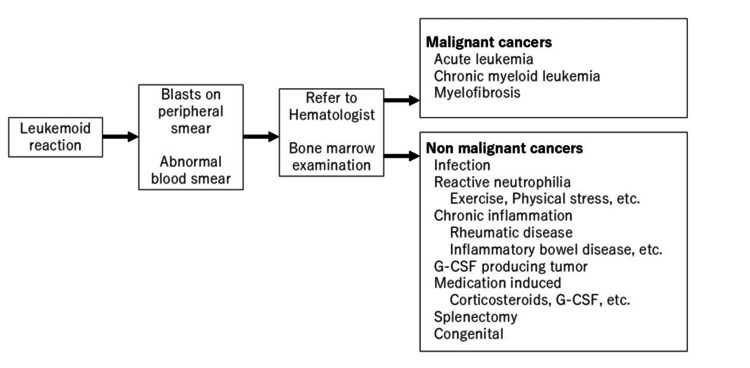
Flowchart of the inclusion process for patients with an increased WBC count G-CSF, granulocyte colony-stimulating factor; WBC, white blood cell

The G-CSF level ranged from 77.1 pg/mL to 4032 pg/mL (normal, 10.5-57.5 pg/mL) in all patients in Table 2. The finding of an elevated G-CS level can indicate a suspected G-CSF-producing bladder carcinoma. Portich et al. reported that the leukemoid reaction was secondary to hematopoietic neoplasms (162 of 267 patients, 60.6%), infection (59 of 267 patients, 22.1%), nonhematopoietic neoplasms (17 of 267 patients, 6.4%: lung cancer (4 patients), pancreatic cancer (4 patients), gastric cancer (2 patients), or other tumors (7 patients)), or other causes (29 of 267 patients, 10.9%) [12]. A common cause for an increased WBC count is hematopoietic neoplasm or infection. However, when a patient has a nonhematopoietic neoplasm and an abnormal number of WBCs, despite the lack of a hematopoietic neoplasm or inflammation, clinicians should be concerned about a G-CSF-producing nonhematopoietic neoplasm. Figure 4 provides a flowchart for patients with an increased WBC count. If clinicians note blasts on a peripheral smear or abnormal blood smear, the clinician should refer the patient to a hematologist and should consider having the patient undergo a bone marrow examination [13]. If malignant cells are detected in a bone marrow examination, malignant cancers, such as acute leukemia, chronic myeloid leukemia, or myelofibrosis, should be suspected. If malignant cancer is excluded, then infection, reactive neutrophilia (exercise, physical stress, etc.), chronic inflammation (rheumatic disease, inflammatory bowel disease, etc.), G-CSF-producing tumor, medication-induced (corticosteroids, G-CSF, etc.), splenectomy, or congenital conditions should be suspected [14].

The chemotherapy regimen for G-CSF-producing advanced/metastatic bladder carcinoma is unclear. Muramatsu-Maekawa et al. reported that they continued to prescribe pembrolizumab to a patient with G-CSF-producing bladder carcinoma for one year as the second-line treatment after the patient had undergone four courses of chemotherapy with a combination of gemcitabine and carboplatin [4]. Their patient had not experienced a recurrence or any adverse event. Takeda et al. report that pembrolizumab resulted in a favorable response in G-CSF-producing upper urinary tract urothelial carcinoma [15]. Miyazaki et al. report administering pembrolizumab as the first-line treatment to six patients with G-CSF-producing lung cancer; they noted that two patients had a partial response, one patient had stable disease, and three patients had disease progression [16]. Pembrolizumab may be useful in the treatment of G-CSF-producing bladder carcinoma. In addition, Galsky et al. report that adjuvant nivolumab is useful for treating patients with high-risk muscle-invasive urothelial carcinoma, based on Checkmate 274, and that pembrolizumab and nivolumab may be beneficial in the treatment of G-CSF-producing bladder carcinoma [17]. Powles et al. reported that enfortumab vedotin and pembrolizumab in untreated advanced urothelial cancer were useful for patients with untreated advanced urothelial cancer, based on EV-302 Study findings [18]. Enfortumab vedotin and pembrolizumab may be available for G-CSF-producing bladder carcinoma.

The prognosis of G-CSF-producing bladder carcinoma is poor. Table 2 shows that two patients died without treatment such as radical cystectomy or chemotherapy within 6 months after the initial visit because of metastasis, one patient died 15 days after the initial chemotherapy because of respiratory failure due to a brainstem infarction, and three patients (including our case) died within 5 months after radical cystectomy because of metastasis and local recurrence.

## Conclusions

Based on this case and a review of the literature, we concluded that G-CSF-producing bladder carcinoma may have a pathologically high-grade and severe clinical course. The treatment of G-CSF-producing bladder carcinoma has not been determined. If a clinician observes a bladder tumor with an abnormal increase in the WBC count, despite the lack of a hematopoietic neoplasm or inflammation, a G-SCF-producing bladder carcinoma should be considered.
